# Race, Ethnicity, Psychosocial Factors, and Telomere Length in a Multicenter Setting

**DOI:** 10.1371/journal.pone.0146723

**Published:** 2016-01-11

**Authors:** Shannon M Lynch, M. K. Peek, Nandita Mitra, Krithika Ravichandran, Charles Branas, Elaine Spangler, Wenting Zhou, Electra D. Paskett, Sarah Gehlert, Cecilia DeGraffinreid, Timothy R. Rebbeck, Harold Riethman

**Affiliations:** 1 University of Pennsylvania, Philadelphia, PA, United States of America; 2 Fox Chase Cancer Center, Philadelphia, PA, United States of America; 3 University of Texas Medical Branch, Galveston, TX, United States of America; 4 Wistar Institute, Philadelphia, PA, United States of America; 5 The Ohio State University, Columbus, OH, United States of America; 6 Washington University, St. Louis, MO, United States of America; 7 Dana Farber Cancer Institute and Harvard University, Boston, MA, United States of America; 8 Old Dominion University, Norfolk, VA, United States of America; University of Tokyo, JAPAN

## Abstract

**Background:**

Leukocyte telomere length(LTL) has been associated with age, self-reported race/ethnicity, gender, education, and psychosocial factors, including perceived stress, and depression. However, inconsistencies in associations of LTL with disease and other phenotypes exist across studies. Population characteristics, including race/ethnicity, laboratory methods, and statistical approaches in LTL have not been comprehensively studied and could explain inconsistent LTL associations.

**Methods:**

LTL was measured using Southern Blot in 1510 participants from a multi-ethnic, multi-center study combining data from 3 centers with different population characteristics and laboratory processing methods. Main associations between LTL and psychosocial factors and LTL and race/ethnicity were evaluated and then compared across generalized estimating equations(GEE) and linear regression models. Statistical models were adjusted for factors typically associated with LTL(age, gender, cancer status) and also accounted for factors related to center differences, including laboratory methods(i.e., DNA extraction). Associations between LTL and psychosocial factors were also evaluated within race/ethnicity subgroups (Non-hispanic Whites, African Americans, and Hispanics).

**Results:**

Beyond adjustment for age, gender, and cancer status, additional adjustments for DNA extraction and clustering by center were needed given their effects on LTL measurements. In adjusted GEE models, longer LTL was associated with African American race (Beta(β)(standard error(SE)) = 0.09(0.04), p-value = 0.04) and Hispanic ethnicity (β(SE) = 0.06(0.01), p-value = 0.02) compared to Non-Hispanic Whites. Longer LTL was also associated with less than a high school education compared to having greater than a high school education (β(SE) = 0.06(0.02), p-value = 0.04). LTL was inversely related to perceived stress (β(SE) = -0.02(0.003), p<0.001). In subgroup analyses, there was a negative association with LTL in African Americans with a high school education versus those with greater than a high school education(β(SE) = -0.11(0.03), p-value<0.001).

**Conclusions:**

Laboratory methods and population characteristics that differ by center can influence telomere length associations in multicenter settings, but these effects could be addressed through statistical adjustments. Proper evaluation of potential sources of bias can allow for combined multicenter analyses and may resolve some inconsistencies in reporting of LTL associations. Further, biologic effects on LTL may differ under certain psychosocial and racial/ethnic circumstances and could impact future health disparity studies.

## Introduction

Telomere DNA consists of long stretches of (TTAGGG) repeat DNA located at the ends of chromosomes[1] and are required for the replication and stability of chromosomes[1]. These repeats naturally shorten with age in all replicating somatic cells[2] due to the inability of the cell to copy the ends of DNA and maintain length over time[3]. Beyond chronological age, telomeres can also shorten prematurely in response to cellular oxidative stress[4, 5]. In normal cells, telomere shortening results in cell senescence or apoptosis[6, 7]. Senescence and apoptosis can function as tumor suppressor mechanisms but can also disrupt normal tissue microenvironments and contribute to aging phenotypes[8–11]. Cells with critically short telomeres that escape apoptosis or senescence [12], and continue to replicate, have unstable genomes and are believed to mark a critical step on the pathway to malignant transformation[2, 13] [14, 15] [4, 16].

Leukocyte telomere length (LTL) has emerged as a potential biomarker of aging, cumulative oxidative stress, and disease, and represents a promising intermediate trait linking chronic cellular stress with disease pathogenesis. Several psychological and social conditions have been associated with both an increase in cellular oxidative stress[5, 17] and subsequent LTL shortening[5, 18–21]. Depression[22], perceived stress[23], and educational attainment[5] are associated with LTL attrition. However, elucidating the complex relationship between psychosocial factors and LTL[24] has been difficult, and inconsistent results have been reported in the literature [5].

While previous studies have demonstrated that membership in certain race/ethnic groups may be associated with a range of socioeconomic and psychosocial factors that could result in shorter LTL [25], namely educational level[5] and perceived stress[23], reports on the effects of race/ethnicity on LTL especially are limited and also inconsistent. Most association studies of LTL have been conducted in female and Non-Hispanic White populations[4, 5]. Studies that include racial/ethnic minorities suggest that Non-Hispanic Whites have shorter LTL compared to African Americans(16, 17) and Hispanics[4]. However, one study suggested that African Americans and Hispanics have shorter LTL than Non-Hispanic Whites[25]. Given the implications for disease prevention, as well as the potential insights into common mechanisms affecting cellular oxidative stress and aging, it is important to better understand both the racial and psychosocial contexts in which changes in telomere biology occur using more diverse samples.

Elucidating the relationship between LTL and social factors can be a challenge. This is because inconsistencies in telomere research might be due in part to differences in study population demographics, laboratory approaches, or selected statistical methods that can often vary both within and across LTL association studies[4]. For instance, older age and male gender are consistent population characteristics associated with shorter LTL[4]. Thus, study populations with higher proportions of older males may report associations with LTL more readily than younger female study samples. [4]. From a laboratory perspective, differences in cell types used to measure telomere length (i.e. buccal, blood leukocyte, tissue), DNA extraction methods[26], and type of telomere length assay can affect the validity and reliability of telomere length measurements [4, 5, 14, 15], and ultimately reported LTL associations[4, 5, 14, 15, 27].

Further, laboratory methods and study population demographics often inform statistical approaches in LTL studies. For instance, the assay selected to measure LTL can affect the reporting and statistical analysis of the LTL outcome variable. Terminal restriction fragment(TRF) assays, known as the gold standard for measuring LTL[27, 28], measure (TTAGGG)n lengths directly by analysis of Southern blots of restriction digests of genomic DNA with frequently-cutting enzymes. Telomere length is reported in terms of the average size of the undigested telomere fragment (which lacks sites for palindrome-dependent restriction enzymes) in base pairs or kilobases(kb) for each leukocyte DNA sample. Quantitative polymerase chain reaction (qPCR), a high-throughput technique often used in large, population-based studies[4, 29, 30], outputs LTL in terms of T/S ratios. Here, a PCR-generated signal that is dependent upon the total (TTAGGG)n content of the sample (T) is compared to the PCR signal from a known gene present only once in the genome (S). T/S ratios of experimental genomic DNA samples are then each compared with those of a reference genomic DNA sample, determined under identical experimental conditions, to arrive at a value describing the telomere content of each unknown sample(24,25). Additionally, some studies account for the potential effects of population characteristics on telomere length outcomes and convert T/S ratios or LTL kb into standardized Z-scores that are adjusted for age and gender[31, 32]. Although T/S ratios and LTL derived from TRF are assumed to be closely correlated(24,25), differences in reported telomere length metrics (e.g. kb, ratios, or Z-scores) can make comparisons across studies difficult, and the implications of using various data transformations and statistical approaches on observed LTL associations has yet to be formally evaluated.

In this study, we use data from a multicenter, multi-racial/ethnic, cross-sectional study to investigate the effect of race/ethnicity and psychosocial factors on a disease-related biomarker. The study sample is comprised of centers that used different LTL laboratory methods and that each contribute different population demographics. The purpose of this analysis is two-fold. First, we conduct a comprehensive investigation of the collective effect of laboratory procedures, study participant characteristics, and statistical measures in order to better understand telomere length associations and any potential inconsistencies in observed associations. Second, once these factors have been considered, we evaluate the effect of race/ethnicity on the relationship between psychosocial factors and telomere length.

## Methods

### Study Sample

Our primary study sample was drawn from three centers: the University of Pennsylvania (Penn), the Ohio State University (OSU), and the University of Texas Medical Branch (UTMB). These centers were originally part of the larger Centers for Population Health and Health Disparities[33] whose main disease focus was on the study of cancer. All study participants were recruited between 2004 and 2012. Each center had its own protocol for recruitment and data collection that has been described previously [34–36], and inclusion/exclusion criteria for each study are listed in **Table 1**. Study participants agreed to donate a blood sample to extract genomic DNA, and they completed a standardized questionnaire at the time of study enrollment. Study participants were followed-up for cancer status. Written informed consent was obtained from all participants, and study protocols were approved by the Institutional Review Boards of the University of Pennsylvania, University of Texas-Medical Branch, and Ohio State University.

**Table 1:** Study Descriptions and Inclusion/Exclusion Criteria.

Center	Original Disease Focus: Primary Race/Gender	Sample size(n = 1510)	Inclusion criteria
Ohio State University(OSU)[34]	Cervical cancer: Non-Hispanic White/ underserved women	111	Women from Appalachia with an intact uterine cervix and corpus, not pregnant, and no history of cervical cancer recruited at time of routine cervical cytology.
University of Pennsylvania Hospital System (UPenn)[36]	Prostate cancer: Non-Hispanic White and African-American/men	101	Male prostate cancer patients from UPenn urology clinics with blood sample.
University of Texas Medical Branch(UTMB)[35]	Stress effects near oil refineries: Non-Hispanic and Hispanic Whites and African American/men and women	1298	Population-based sample of Non-Hispanic households and a strata sample of Hispanic households in Texas City, TX.

### Covariates

Variables common to all 3 centers included: gender(male/female), age at enrollment (continuous); race/ethnicity(White/Non-Hispanic, African American/Non-Hispanic, and Hispanic), educational status (less than high school or less than 12 years of schooling; high school education or 12 years of schooling/GED); >high school education or >12 years of schooling), disease status (cancer; yes/no), as well as other behavioral factors, including smoking status (ever/never). The psychosocial factors in this study were defined by perceived stress and depression. To evaluate stress, we used the validated perceived stress scale (PSS)[37, 38]. This is a 10-item global measure of perceived stress where higher scores indicate greater perceived stress(total score range: 1–40). Total PSS was normally distributed in this sample, and we dichotomized this variable to compare high (above median) to low (below median) stress[39, 40]. Questions from the validated Center for Epidemiological Studies-Depression (CES-D) scale[41] and the CES-D revised(R) scale[42] were used to ascertain depressive symptoms. Both the CES-D and CES-DR are 20-item scales (total score range: 0–60). Higher scores, particularly those above 16, suggest more depressive symptoms[41]. The combined total scores from CES-D and CES-DR were positively skewed; we dichotomized at the clinical cut-point of 16[41] to compare those with higher and lower levels of depressive symptoms. PSS, CES-D, and CES-DR scales have been validated in multiethnic studies [43, 44].

### Laboratory/Statistical Methods

Prior to assessing our primary data, we undertook a review of multicenter association studies of LTL in order to ascertain laboratory factors and statistical approaches that appear to contribute to inconsistent LTL associations in a multicenter setting(S1 Protocol; S1 Table) (4):

#### Tissue Source for DNA

All centers followed the same standardized blood draw protocol and used the same tissue source to extract DNA, peripheral blood leukocytes. Twenty milliliters of blood were drawn from each subject by a trained phlebotomist. Samples were centrifuged and buffy coats were stored at -70°C until DNA extraction and telomere assay.

#### DNA Extraction

Genomic DNA was extracted from each center individually and sent to the Wistar Institute for analysis. OSU and UTMB samples were processed using the QIAamp DNA Extraction Kit (Valencia, CA). Penn DNA samples were extracted using Chemagen Magnetic Bead technology (n = 61) and phenol-chloroform extraction (n = 40).

#### Terminal Restriction Fragment (TRF) assay

TRF length assays, also known as Southern Blots, were used to measure LTL from extracted DNA on all study samples (using duplicate samples), as described previously by Kimura et al[45] and detailed in Supplementary Laboratory Methods(S2 Protocol). Briefly, genomic DNA samples were digested with restriction enzymes *Hinf I* (10U) and *Rsa I* (10U; Roche), and mean LTL in kb was determined using Telorun software[45]. All TRF assays were conducted in the same laboratory at the Wistar Institute.

#### Quantitative Telomere PCR (qPCR)

For a subset of Cross-Center samples (Penn, n = 101 and OSU, n = 111), LTL was also measured using the quantitative PCR method developed by Cawthon, modified for compatibility with the Applied Biosystems 7900 HT instrument [30](S2 Protocol). Assays were carried out in triplicate, and center samples were batch analyzed to minimize inter-assay variation. The T/S ratios of each experimental sample relative to the reference sample were generated using the comparative CT (cycle threshold) method[30]. T/S ratios and LTL kb were compared for quality control comparisons.

#### Coefficient of Variation Percentages (CV%)

CV% were calculated for duplicate (TRF measurements) or triplicate(qPCR measurements) samples using the pooled standard deviation of the duplicates or triplicates divided by the overall mean of all measurements. The TRF overall CV was 1.25%. The qPCR intra- and inter-plate CV% were 4.9% and 12.9%, respectively.

#### Statistical Analysis

Data quality control measures were undertaken to identify any potential measurement errors or inconsistencies. Box plots of LTL measurements were generated to identify outlier points or data errors. LTL is described using means, medians, standard deviations and ranges. Distributions of LTL were not normal, and data transformations were conducted for statistical analysis. Methods used in past multicenter studies were used to investigate inconsistencies and LTL associations (S1 Protocol; S1 Table). Specifically, we evaluated correlations between log-transformed LTL from TRF and log-transformed LTL from qPCR measurements in the combined study population, by center, and by DNA extraction method[26] using[4, 27–30] linear regression.

Relevant study population characteristics overall and by center are summarized by medians and frequencies. Comparisons of population characteristics across center and by LTL were conducted using nonparametric tests (Kruskal-Wallis and Wilcoxon ranked sum) for primary evaluation of population demographics, which included comparison of findings related to age and gender to those reported in literature.

Associations between LTL and age, LTL and race/ethnicity, and LTL and psychosocial factors, including education, perceived stress, and depression, were assessed using the two common telomere length metrics reported in multicenter settings (S1 Table), log-transformed telomere length(kb) and LTL Z-score. Inverse-weighted variance Z-scores were calculated by subtracting the log-transformed LTL sample mean from the original sample values and then dividing by the sample standard deviation[31, 32]. Z-scores were also adjusted for age, gender, and cancer status by estimates *within* strata and then taking the weighted average *across* strata[46–51] [52, 53]. Population demographic variables, age, gender, and cancer status, relate to LTL in literature (4) (S1 Protocol/S1 Table). Multivariable linear models and generalized estimating equations((GEE) (using an independence correlation structure and robust standard errors) [54]) were first used to assess associations of LTL with more established population risk factors, age, gender, and cancer status, in order to serve as a quality control check of our data. These GEE and linear regression models of LTL and established risk factors also accounted for potential effects by center and by laboratory methods that are specific to this study sample. After accounting for potential sources of bias, relevant population and laboratory factors were chosen for final inclusion in GEE and linear regression models evaluating associations among LTL, psychosocial factors, and race/ethnicity using stepwise forward and backward variable selection approaches, with a liberal variable inclusion cut-off of p<0.25. Interactions between age, gender, race/ethnicity, and psychosocial factors were then evaluated using appropriate cross-product terms within statistical models. Subgroup analyses were further conducted by race/ethnicity, and in those without cancer and within the UTMB cohort. All P-values were two-sided. All statistical analyses were conducted using STATA version 9.1.

## Results

### Laboratory Methods Evaluation

**Fig 1:**
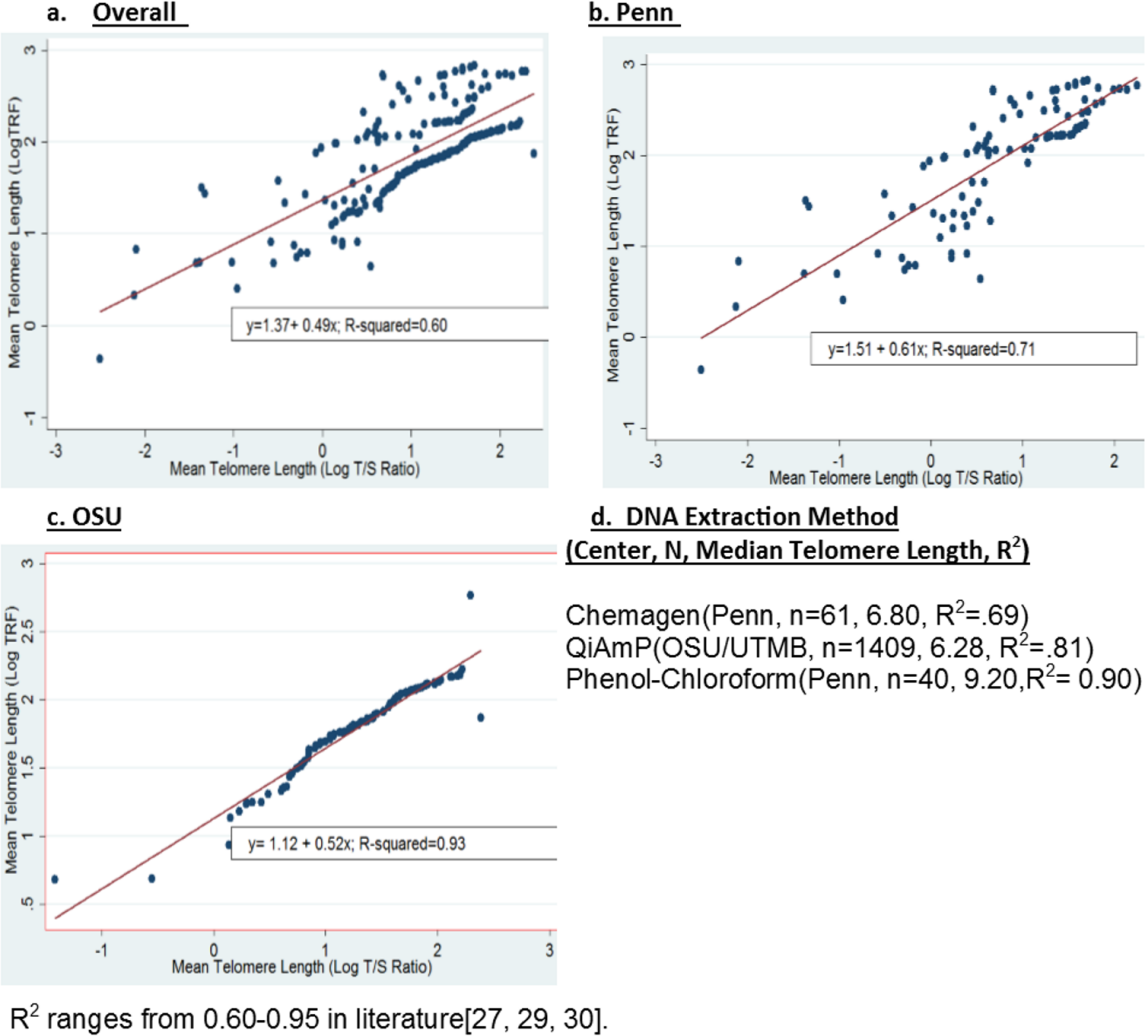
Laboratory Methods Evaluation Comparing Telomere Length by Telomere Assay Type, Center, and DNA extraction method. **a-c**. Comparison of Log-transformed telomere length (LogTRF) from Southern Blot (also known as terminal restriction fragment (TRF) assays) to log-transformed telomere content (T) to standard gene (S) ratios (Log T/S ratio) from quantitative PCR in the overall sample (**a**) and by center (**b,c**; where b = University of Pennsylvania (Penn); c = Ohio State University(OSU)). **d**. Median telomere length from terminal restriction fragment assays and correlation or R^2^ by DNA extraction method.

Laboratory factors affecting LTL measures in previous studies were identified (i.e. type of telomere assay, comparison of telomere assays, DNA extraction protocols, CV%) and evaluated for effects on LTL measurements in our study. In pilot experiments, TRF assays consistently yielded excellent measurement CVs <2% across study centers. qPCR measurements often have higher CV%s compared to TRF assays, which was consistent here (Penn: qPCR 12.0 CV% and TRF 0.93 CV%; OSU: qPCR 1.2 CV% and TRF 0.01 CV%; UTMB: qPCR 27 CV% and TRF 1.9 CV%) [14]. UTMB qPCR samples yielded an unacceptably high measurement CV%, possibly due to an unknown analyte affecting the qPCR reaction (24)). Thus LTL measured from TRF assays served as our main outcome variable, and UTMB qPCR samples were excluded from additional quality control checks. qPCR and TRF(Southern Blot) quality control comparisons were made with OSU and Penn samples(n = 211)[14]. The relationship between log-transformed TRF measures and log-transformed T/S ratios showed an overall R^2^ of 0.60(**Fig 1A**). The R^2^ within centers was 0.71 for Penn (**Fig 1B**) and 0.93 for OSU (**Fig 1C**). Comparing log-transformed TRF to log-transformed T/S ratios by DNA extraction method, the R^2^ for QiAmp DNA extraction was 0.81; for Chemagen, 0.69 and for phenol-chloroform, 0.90. The mean (standard deviation) LTL from TRF across all centers was 6.55kb (2.86). Within center, mean LTL was 8.42kb (4.50) for Penn; 6.34kb (1.95) for OSU; and 6.42kb (2.71) for UTMB. Median LTL was significantly different by extraction method (p-value<0.001) (**Fig 1D**). However, median LTLs were not significantly different between Qiagen and Chemagen methods (p-value = 0.48).

#### Study demographic evaluation

Baseline characteristics of the study overall (n = 1510) and by center were evaluated to determine potential clustering and confounding effects by center (**Table 2**). The overall study population was 58.8% female. 15.7% had a cancer diagnosis, and 51% had ever smoked cigarettes. The average age was 50.6 years, with a standard deviation of 15.6. All population characteristics, except for smoking, were significantly different across centers. Baseline study population characteristics of the combined study population (includes all 3 centers) were compared on median LTL and log-transformed LTL(kb) in order to compare our results to literature and to identify factors related to LTL that could be tested in forward and backward regression models with age and LTL(**Table 3).** Only cancer status had a significant association with median LTL; cancer cases had longer LTL than those without cancer (p-value = 0.02). There was no statistical relationship between LTL and gender (median LTL(kb), interquartile range (IQR): men = 6.43, 4.14–8.39; women = 6.33, 4.39–8.27; Table 3) in the overall cohort and when restricting the population to those without cancer(median TRF(kb), IQR: men = 6.01kb, 4.10–8.00; women = 6.33, 4.48–8.27, p-value = 0.12; Supplementary Table 2**)**.

**Table 2:** Study Characteristics.

	ALL Centers	Penn	OSU	UTMB	p-value^b^
**Population Characteristics**					
Total Population (n)	1510	101	111	1298	
Median Telomere length (kb)^a^	6.4 (4.3–8.3)	8.7 (4.2–11.8)	6.3 (5.1–7.9)	6.3 (4.2–8.3)	0.0001
Median Age^a^	51 (38–63)	58 (53–63)	30 (26–43)	51 (38–64)	0.0001
Male Gender (%)	41.2	100	0	40.1	0.0001
Cancer diagnosis(%)	15.7	100	0	10.5	<0.001
Ever Cigarette Smokers(%)	51.0	57.4	42.3	51.2	0.08
Race (%)					
Non-Hispanic White	45.6	89.0	98.2	37.8	
African American	9.4	11.0	1.8	9.9	
Hispanic	45.0	0	0	52.3	<0.001
Education(%)					
> High School	37.0	72.3	64.9	31.8	
High School/GED	29.8	23.7	23.4	30.8	
<High School	33.2	4.0	11.7	37.4	<0.001
**Psychosocial Factors**					
Median Total Depression Score	6(1–15)	8 (3–13)	12 (7–22)	5 (0–14)	0.0001
Median Total Perceived Stress	19 (17–22)	20 (18–22)	22 (19–24)	19(16–22)	0.0001

Abbreviations: University of Pennsylvania(Penn), Ohio State University(OSU), University of Texas Medical Branch(UTMB)

^a^ Medians(interquartile range for the median)

^b^ p-values comparing characteristics across each of the 3 centers using Kruskal Wallis Test or Fisher’s Exact Test. Range from low to high for Depression (0–60) and Perceived Stress Scale (0–40).

**Table 3:** Unadjusted Median Telomere Length(kb) and Mean Log-Telomere Length(TL in kb) by Study Characteristics(ALL n = 1510).

	Median TL (kb) (Interquartile Range)^a^	p-value^b^	Mean logTL(SD)	p-value^c^
**Population Characteristics**				
**Age**				
Younger age(< = 51)	6.39(4.33–8.29)		1.78(0.44)	
Older age(>51)	6.30(4.20–8.32)	0.68	1.77(0.50)	0.51
**Gender**				
Female	6.33 (4.39–8.27)		1.77(0.44)	
Male	6.43 (4.14–8.39)	0.58	1.78(0.51)	0.07
**Cancer diagnosis**				
Yes	7.14 (4.00–9.18)		1.83(0.58)	
No	6.28 (4.30–8.17)	0.02	1.77(0.45)	0.08
**Ever Smoker**				
**Yes**	6.45(4.25–8.29)	0.71	1.78(0.47)	
**No**	6.28(4.31–8.31)		1.77(0.48)	0.89
**Race**				
Non-Hispanic White	6.11(4.19–8.23)		1.75(0.490	
African American	6.61 (4.56–8.82)		1.83(0.48)	
Hispanic	6.42 (4.42–8.27)	0.12	1.79(0.45)	0.18
**Education**				
> High School	6.35(4.29–8.30)		1.77(0.50)	
High School/GED	6.13(4.29–8.30)		1.76(0.48)	
<High School	6.43(4.53–8.15)	0.61	1.80(0.43)	0.46
**Psychosocial Factors**				
**Total Perceived Stress**				
High Stress (>19)	6.33(4.20–8.32)		1.77(0.48)	
Low Stress (< = 19)	6.39(4.33–8.26)	0.60	1.78(0.47)	0.69
**Total Depression**				
High Depression (>16)	6.45(4.50–8.52)		1.81(0.44)	
Low Depression (< = 16)	6.33(4.24–8.23)	0.12	1.76(0.48)	0.19

Ranges of Median and Mean telomere length are similar to those reported in literature[27, 29, 30].

^a^ Medians(interquartile range for the median)

^b^ p-values comparing characteristics across 3 or more groups using Kruskal Wallis Test, otherwise used Wilcoxon Ranked Sum Test

^c^ p-values comparing characteristics across 3 or more groups using ANOVA, otherwise used T-test.

#### Associations between population demographics, laboratory methods and LTL

There was no correlation between age and log-transformed LTL(R^2^ = -0.08, p-value = 0.45) and Z-score LTL(R^2^ = -0.10, p-value = 0.49) in crude models. In adjusted models, the best fitting linear regression and GEE model for continuous age and log-transformed LTL or Z-score were the same and included the following: gender (GEE p-value<0.001), cancer status (GEE p-value<0.001), a gender-cancer status interaction (GEE p-value<0.001), and DNA extraction method (GEE p-value<0.001). The gender-cancer status interaction remained when using OSU/UTMB(p-value = 0.01) or UTMB data only(p-value = 0.02). Thus, investigations of LTL, race/ethnicity, and psychosocial factors in the full study sample included adjustments for these model variables.

#### Association of Race/Ethnicity, Psychosocial Factors and LTL

The distribution of psychosocial factors and race/ethnicity differed significantly across centers (**Table 2**). The sample was comprised of 45.6% non-Hispanic Whites, 45.0% Hispanics, and 9.4% African Americans. The Penn and OSU study participants reported higher levels of education than UTMB (p-value<0.001). OSU, which included only females, reported the highest levels of stress and depression (p-value = 0.03). There were no statistically significant differences in mean or median LTL across psychosocial variable groups (**Table 3**). However, African Americans had the longest median LTL (6.61kb, IQR = 4.56–8.82), and Non-Hispanic Whites the shortest (6.11kb, IQR = 4.19–8.23). Patterns were consistent when restricting the study population to those without cancer (S2 Table) and UTMB only. No significant interactions between population demographics and psychosocial factors were observed.

Associations with log-transformed LTL and LTL Z-scores with race/ethnicity and psychosocial factors were estimated using both adjusted linear regression (Model 1) and GEE models (Model 2)(**Table 4).** Regardless of LTL measure or statistical model, there was a significant, direct relationship between LTL and race/ethnicity. For both LTL outcome measures (log-transformed LTL and Z-score), GEE models presented a significant relationship between lower levels of education (less than high school) (log-transformed LTL p-value = 0.02) and higher levels of perceived stress(log-transformed p-value<0.001). Results were similar when analyzing perceived stress and depression as continuous variables (data not shown). Reported associations between race/ethnicity and psychosocial factors were similar when limiting the study population to those without cancer (which eliminated adjustments for cancer status and the interaction of gender and cancer status) and UTMB only (which eliminated adjustment for center and laboratory effects). Since both LTL measures resulted in similar association results, and there are clustering effects by center, the best model for this data appears to be GEE models analyzed with log-transformed LTL.

**Table 4:** Adjusted Regression Estimates(Standard Errors) of Individual Race/Ethnicity, Education, Psychosocial Factors and Log-Transformed and Z-Score Telomere Length(TL in kb).

	Log-Transformed TL		Z-Score TL	
	Model 1	Model 2	Model 1	Model 2
Race (compared to Non-Hispanic Whites)				
African-American	0.10(0.04)++	0.09(0.04)++	0.19(0.09)++	0.17(0.08)++
Hispanic	0.07(0.03)++	0.06(0.01)+++	0.16(0.06)++	0.13(0.03)+++
Education (compared to > High School)				
High School education	0.01(0.03)	0.01(0.03)	0.01(0.06)	0.0004(0.04)
Less than high school	0.06(0.03)+	0.06(0.02)++	0.13(0.06)+	0.12(0.04)++
Perceived Stress				
High Stress(compared to low stress)	-0.02(0.02)	-0.02(0.003)+++	-0.05(0.05)	-0.05(0.003)+++
Depression				
High Depression (compared to low)	0.04(0.03)	0.04(0.02)+	0.07(0.06)	0.07(0.05)+

**Model 1** = Linear Regression; **Model 2** = GEE, accounts for clustering of center. LogTL model adjusted by age, gender, cancer status, DNA extraction, and the interaction of gender and cancer status. Z-score adjusted by age, gender and cancer status and model adjusted by DNA extraction.

+++p-value ≤ 0.001

++p-value >0.001 and <0.05

+suggestion of significance p-value<0.15, but ≥0.05.

No statistically significant associations between log-transformed LTL and psychosocial factors were reported for Caucasians or Hispanics using GEE models(**Table 5**). Compared to those with more than a high school education, having only a high school education was significantly related to shorter LTL (p-value<0.001) in African Americans.

**Table 5:** GEE Estimates(Standard Errors) of Individual Socioeconomic and Psychosocial Factors and Log-Transformed Telomere Length(TL in kb) stratified by Race/Ethnicity^a^.

	Non-Hispanic Whites (n = 688)	African Americans(n = 142)	Hispanics^b^(n = 688)
Education (compared to > high school education)			
High School education	0.03(0.04)	-0.11(0.03)+++	0.001(0.05)
Less than high school	-0.02(0.05)	0.01(0.02)	0.07(0.04)+
Perceived Stress (compared to low stress)			
High Stress	0.02(0.03)	-0.001(0.01)	-0.05(0.04)+
Depression (compared to low)			
High Depression	0.09(0.05)	0.02(0.04)	-0.01(0.04)

^a^ Model adjusted for age, gender, cancer status, gender-cancer status interaction, and DNA extraction method

^b^Linear Regression Model is reported since all Hispanics come from only 1 center. This model is adjusted by age, gender, cancer status and the interaction of gender and cancer status.

+++p-value ≤ 0.001

++p-value >0.001 and <0.05

+suggestion of significance p-value<0.15, but ≥0.05.

## Discussion

Inconsistent associations between LTL, race/ethnicity, and psychosocial factors in literature have been reported [5] (16, 17) [23] [25], and few studies have evaluated the association between LTL and psychosocial factors within race/ethnic subgroups[25]. Inconsistencies in literature between socioeconomic and psychosocial factors and LTL have been attributed to different laboratory and statistical approaches employed in these telomere studies[4], but few studies have evaluated these methodological effects. Multi-center studies serve as an ideal opportunity for evaluating methodological effects on LTL associations since they often combine data from centers with heterogeneous populations and varying laboratory approaches. Our findings suggest that combining and comparing data from multiple centers is valid and can have little effect on LTL associations. Inconsistencies can be minimized through proper evaluation of factors that could influence LTL measurements and with appropriate statistical adjustments.

We first assessed our laboratory methods and demonstrated that our telomere measurements in the combined study population were reliable and valid compared to other published studies[4, 5, 14, 15]. More specifically, we evaluated the source of DNA, type of telomere length assays, CV percents, and DNA extraction techniques[4, 5, 14, 15] since they are known to contribute to discrepancies in reporting associations between LTL and disease[3, 14, 26]. Choice of tissue type (blood leukocytes) and assay (Southern Blot) in our study were consistent with those used in literature(4), and correlations(R^2^) between TRF(Southern Blot) LTL and T/S ratios for Penn and OSU were within range of other studies (0.60–0.95) [27, 29, 30, 52, 53]. We would expect our results to be similar in multicenter studies that use qPCR approaches (and a single laboratory to measure LTL), given that quality control checks like CV% and correlations between Southern Blot and qPCR generally have satisfactory ranges (i.e. R^2^ from 0.60 to 0.95) [27, 29, 30, 52, 53]. OSU had higher R^2^ values than Penn, which could potentially be explained by sample purity. qPCR is more sensitive to analytes than TRF(28, 29, 30), and Penn samples could have contained more analytes. Thus, the use of LTL from TRF as the main outcome variable in our analysis was appropriate.

Similar to published studies where qPCR was used, phenol-chloroform DNA extraction resulted in longer mean and median telomeres than Qiagen methods[14, 26], and Qiagen and Chemagen, both column-based extraction methods, yielded similar median LTL results [14, 26] in our study, which usedTRF assays. Thus, the relationship between qPCR and Southern Blot LTL was likely not affected by DNA extraction method. However, given that the majority of our samples were extracted using Qiagen and Chemagen(97.3%), our LTL measurements could be underestimated and result in Type II error. However, the bias is likely nondifferential. Few multicenter studies of LTL report and consider the effects of DNA extraction on study outcomes(S1 Table), and DNA extraction appears to contribute to inconsistent findings in telomere association studies[14, 26].

We also assessed population demographic effects or established risk factor effects on LTL in our sample. Age and male gender have been associated with shorter LTL(4) in many studies. While we see the same trends in our data, we do not observe statistically significant associations in crude, single variable analyses(S2 Table). Although the linear relationship between age and LTL was weaker in the present study for log-transformed LTL(R^2^ = -0.08) than previously reported(R^2^~0.15 [4]), the attenuated association observed between age and LTL when adjusting for other covariates, like gender, is consistent with other studies[53]. Additionally, the rate of telomere attrition may vary over lifespan, with some studies suggesting more rapid attrition in younger ages (childhood) and in later decades of life (over age 70) [55, 56]; the age range of the sample was 26–64 and the median age of the sample was relatively young at 51 years (Table 2). We also found that male cancer cases had longer telomeres compared to non-cancer cases, and this has been observed in literature[57, 58], although inconsistently[59].

These initial evaluations informed which laboratory and population factors may affect LTL associations with race/ethnicity and psychosocial factors in our study. DNA extraction method, along with age, gender, cancer status, and the interaction of gender and cancer status, were significant confounders. Center-specific study recruitment led to specialized groupings of gender and cancer status by center. Thus, center was a cluster variable, and GEE models, which accounted for the within and between effects of the center cluster variable and include stricter standard errors[60], appeared more appropriate in our analyses. Few multi-center association studies of LTL have accounted for potential cluster effects (S1 Table), and we found no significant associations between LTL, race/ethnicity, and psychosocial factors in crude models without adjustment for clustering effects, laboratory methods, or relevant population demographics in our multicenter sample. This finding and concern over additional variability in LTL in those with cancer and by center prompted us to compare findings when restricting the population to those without cancer and UTMB only (where both center effects and differences in DNA extraction would not be a concern). We found that results were robust and that extraneous variability in LTL appeared to be removed with adjustment for relevant population and laboratory methods. This consistency across study populations suggests that consistent associations could be realized with proper adjustments, while our crude analyses suggest that associations could be missed without accounting for potential sources of bias.

We also evaluated the choice of outcome measure (i.e., log-transformed LTL or Z-score). Most multicenter studies of LTL report log-transformed LTL (S1 Table). However, Z-scores standardize telomeres based on sample distributions and may be more appropriate in instances where the distribution of LTL greatly differs by center or when confounders or model adjustment variables differ by center. Although the magnitude of effects appear different (and often higher with Z-score), they are not comparable. This is because the data transformation associated with each of these measures lends itself to different interpretations. For instance, log-transformed LTL describes changes in log-transformed LTL and Z-score describes changes in LTL standard deviations. Nevertheless, patterns of association between LTL and race/ethnicity and psychosocial factors were similar regardless of which telomere outcome measure (log-transformed LTL or Z-score) was used.

To our knowledge, this is the first study to evaluate the main effects of race/ethnicity and psychosocial factors on LTL, as well as the effect of race/ethnicity on the relationship between socioeconomic and psychosocial factors and LTL. It is also the first study to more comprehensively investigate the collective effect of laboratory procedures, study population characteristics, and statistical measures on reported LTL associations. We found significant associations between LTL and race/ethnicity, longer LTL and low levels of education, and shorter LTL and higher levels of perceived stress. Associations between high levels of perceived stress and shorter LTL have been reported(5). We are only the second study to report that both African Americans and Hispanics have longer LTLs than Non-Hispanic Whites(4). Having less than a high school education was associated with longer LTL, which is an association not typically reported in literature(5)[25]. When stratifying the analysis by race, there was a suggested association between longer LTL and less than a high school education for Hispanics, and a significant association between shorter LTL and having a high school education for African Americans, where greater than a high school education was the reference group. Thus, the racial, ethnic and educational composition of our sample (including a large number of Hispanics with low education) may have affected our education findings. Studies have found correlations with socioeconomic status (SES) related to education and income, and race, namely lower SES conditions are associated with African Americans[61]. Being Hispanic is also associated with lower levels of education in literature, as well as improved mortality rates compared to African Americans[61], referred to as the Hispanic paradox[62–64]. Given that shorter LTL is believed to be related to mortality[4], racial composition appears to be an important consideration in LTL studies.

Our study had some limitations. This was a cross-sectional investigation, limiting us to studying variables that were common to all 3 centers. For instance, duration and severity of depression and perceived stress are more consistently associated with shorter LTL[65], and LTL is likely to shorten over time(1). Stratified analyses by race yielded small samples, particularly for African Americans, but findings suggest studies focused on telomere biology by race/ethnicity are warranted. Like most LTL association studies, differences in mean LTL could be influenced by the proportions of different kinds of leukocytes[66]. The average LTL in any given study is considered to be a general average of all the LTLs across all chromosomes and blood leukocytes. Although it is unclear whether differential cell counts are affected by race/ethnicity in a way that would explain the patterns we observed, one previous study found no association between leukocyte type and LTL in African Americans or Non-Hispanic Whites[67].

The large multi-ethnic and multicenter composition of our study allowed for more in depth analysis of the effects of laboratory and statistical approaches on telomere length associations. Our study demonstrated that with proper evaluation and adjustment of center and laboratory effects, combining data from multiple centers, with different laboratory approaches and population characteristics, can be a powerful and valid approach for assessing LTL associations. In addition, evaluating methodological effects, similar to what we have done here, within and across LTL studies may help resolve inconsistent reports of LTL associations. Our data provide evidence of an association between Hispanics and African Americans and longer LTLs, as well as potential relationships between educational level, perceived stress and LTL for certain racial/ethnic sub-groups. Further study into the effects of socioeconomic and psychosocial factors on LTL by race/ethnicity could have implications for research involving health disparities and disease outcomes.

## Supporting Information

S1 Protocol Review of Multilevel studies in Telomere Length. (DOCX)

S2 Protocol Supplementary Laboratory Methods. (DOCX)

S1 Table Evaluation and Summary of Methodologies employed in Multi-center, Telomere Length(TL) Association Studies from 2002-Present. (DOCX)

S2 Table Unadjusted Median and Mean LogTelomere Length(TL in kb) by Study Characteristics(No Cancer, n = 1261). (DOCX)

## References

[1] Cheung ALM, Deng W. Telomere dysfunction, genome instability and cancer. Frontiers in Bioscience. 2008;13(6):2075–90. 17981693 10.2741/2825

[2] Londoño-Vallejo JA. Telomere instability and cancer. Biochimie. 2008;90(1):73–82. 17728038 10.1016/j.biochi.2007.07.009

[3] Blackburn EH. Telomere states and cell fates. Nature. 2000;408(6808):53–6. 11081503 10.1038/35040500

[4] Sanders JL, Newman AB. Telomere Length in Epidemiology: A Biomarker of Aging, Age-Related Disease, Both, or Neither? Epidemiologic Reviews. 2013;35(1):112–31. 10.1093/epirev/mxs008 23302541 PMC4707879

[5] Starkweather AR, Alhaeeri AA, Montpetit A, Brumelle J, Filler K, Montpetit M, et al An Integrative Review of Factors Associated with Telomere Length and Implications for Biobehavioral Research. Nursing Research. 2014;63(1):36–50 10.1097/NNR.0000000000000009 24335912 PMC4112289

[6] Palm W, de Lange T. How shelterin protects mammalian telomeres. Annu Rev Genet. 2008;42:301–34. Epub 2008/08/06. 10.1146/annurev.genet.41.110306.130350 . 18680434

[7] Muñoz P, Blanco R, Blasco MA. Role of the TRF2 Telomeric Protein in Cancer and Aging. Cell Cycle. 2006;5(7):718–21. 16582635 10.4161/cc.5.7.2636

[8] Coppé JP, Desprez P.Y., Krtolica A, Campisi J. The senescence-associated secretory phenotype: the dark side of tumor suppression. Annu Rev Pathol. 2010;5:99–118. 10.1146/annurev-pathol-121808-102144 20078217 PMC4166495

[9] Davalos AR, Coppe JP, Campisi J, Desprez PY. Senescent cells as a source of inflammatory factors for tumor progression. Cancer Metastasis Rev. 2010;29:273–83. 10.1007/s10555-010-9220-9 20390322 PMC2865636

[10] Jaskelioff M, Muller FL, Paik JH, Thomas E, Jiang S, Adams AC, et al Telomerase reactivation reverses tissue degeneration in aged telomerase-deficient mice. Nature. 2010;469:102–6. 10.1038/nature09603 21113150 PMC3057569

[11] Sahin E, Depinho RA. Linking functional decline of telomeres, mitochondria and stem cells during ageing. Nature 2010;464:520–8. 10.1038/nature08982 20336134 PMC3733214

[12] Tea Halvorsen. Telomerase Activity Is Sufficient To Allow Transformed Cells To Escape from Crisis. Mol Cell Biol. 1999;19(3):1864–70. 10022873 10.1128/mcb.19.3.1864 PMC83979

[13] Koorstra JBM, Hustinx SR, Offerhaus GJA, Maitra A. Pancreatic Carcinogenesis. Pancreatology. 2008;8(2):110–25. 10.1159/000123838 18382097 PMC2663569

[14] Cunningham JM, Johnson RA, Litzelman K, Skinner HG, Seo S, Engelman CD, et al Telomere Length Varies By DNA Extraction Method: Implications for Epidemiologic Research. Cancer Epidemiology Biomarkers & Prevention. 2013;22(11):2047–54. 10.1158/1055-9965.epi-13-0409 PMC3827976 24019396

[15] Wentzensen IM, Mirabello L, Pfeiffer RM, Savage SA. The Association of Telomere Length and Cancer: a Meta-analysis. Cancer Epidemiology Biomarkers & Prevention. 2011;20(6):1238–50. 10.1158/1055-9965.epi-11-0005 PMC3111877 21467229

[16] Butt HZ, Atturu G, London NJ, Sayers RD, Bown MJ. Telomere Length Dynamics in Vascular Disease: A Review. European journal of vascular and endovascular surgery: the official journal of the European Society for Vascular Surgery. 2010;40(1):17–26. 10.1016/j.ejvs.2010.04.012 20547081

[17] Shalev I, Entringer S, Wadhwa PD, Wolkowitz OM, Puterman E, Lin J, et al Stress and telomere biology: A lifespan perspective. Psychoneuroendocrinology. 2013;in press. 10.1016/j.psyneuen.2013.03.010 PMC3735679 23639252

[18] Epel ES, Lin J, Dhabhar FS, Wolkowitz OM, Puterman E, Karan L, et al Dynamics of telomerase activity in response to acute psychological stress. Brain Behav Immun. 2010;24:531–9. 10.1016/j.bbi.2009.11.018 20018236 PMC2856774

[19] O’Donovan A LJ, Dhabhar FS, Wolkowitz O, Tillie JM, Blackburn E, Epel E. Pessimism correlates with leukocyte telomere shortness and elevated interleukin-6 in post-menopausal women. Biol Psychiatry. 2009;24:446–9. 10.1016/j.bbi.2008.11.006 PMC2719778 19111922

[20] O’Donovan A, Epel E, Lin J, Wolkowitz O, Cohen B, Maguen S, et al Childhood trauma associated with short leukocyte telomere length in posttraumatic stress disorder. Biol Psychiatry. 2011a;70:465–71. 21489410 10.1016/j.biopsych.2011.01.035 PMC3152637

[21] O’Donovan A, Tomiyama J, Lin J, Puterman E, Adler NE, Kemeny M, et al Stress appraisals and cellular aging: A key role for anticipatory threat in the relationship between psychological stress and telomere length. Brain, Behavior, and Immun. 2012. 10.1016/j.bbi.2012.01.007 PMC3322317 22293459

[22] Wolkowitz OM, Mellon SH, Epel ES, Lin J, Dhabhar FS, Su Y, et al Leukocyte Telomere Length in Major Depression: Correlations with Chronicity, Inflammation and Oxidative Stress—Preliminary Findings. PLoS ONE. 2011;6(3):e17837 10.1371/journal.pone.0017837 21448457 PMC3063175

[23] Parks CG, Miller DB, McCanlies EC, Cawthon RM, Andrew ME, DeRoo LA, et al Telomere Length, Current Perceived Stress, and Urinary Stress Hormones in Women. Cancer Epidemiology Biomarkers & Prevention. 2009;18(2):551–60. 10.1158/1055-9965.epi-08-0614 PMC2696885 19190150

[24] Epel ES, Blackburn EH, Lin J, Dhabhar FS, Adler NE, Morrow JD, et al Accelerated telomere shortening in response to life stress. Proc Natl Acad Sci. 2004;101:17312–5. 15574496 10.1073/pnas.0407162101 PMC534658

[25] Diez Roux AV, Ranjit N, Jenny NS, Shea S, Cushman M, Fitzpatrick A, et al Race/ethnicity and telomere length in the Multi-Ethnic Study of Atherosclerosis. Aging Cell. 2009;8(3):251–7. 10.1111/j.1474-9726.2009.00470.x 19302371 PMC2713110

[26] Hofmann J, Hutchinson AA, Cawthon R, Liu CS, Lynch SM, Lan Q, et al. Telomere Length Varies By DNA Extraction Method: Implications for Epidemiologic Research–Letter. Unpublished. 2014. 10.1158/1055-9965.EPI-14-0145 PMC4051398 24798729

[27] Aviv A, Hunt SC, Lin J, Cao X, Kimura M, Blackburn E. Impartial comparative analysis of measurement of leukocyte telomere length/DNA content by Southern blots and qPCR. Nucleic Acids Research. 2011;39(20):e134 10.1093/nar/gkr634 21824912 PMC3203599

[28] Elbers CC, Garcia ME, Kimura M, Cummings SR, Nalls MA, Newman AB, et al Comparison Between Southern Blots and qPCR Analysis of Leukocyte Telomere Length in the Health ABC Study. The Journals of Gerontology Series A: Biological Sciences and Medical Sciences. 2013 10.1093/gerona/glt121 PMC4049151 23946336

[29] Cawthon RM. Telomere measurement by quantitative PCR. Nucleic Acids Research. 2002;30(10):e47 12000852 10.1093/nar/30.10.e47 PMC115301

[30] Cawthon RM. Telomere length measurement by a novel monochrome multiplex quantitative PCR method. Nucleic Acids Research. 2009;37(3):e21 10.1093/nar/gkn1027 19129229 PMC2647324

[31] Du M, Prescott J, Kraft P, Han J, Giovannucci E, Hankinson SE, et al Physical Activity, Sedentary Behavior, and Leukocyte Telomere Length in Women. American Journal of Epidemiology. 2012;175(5):414–22. 10.1093/aje/kwr330 22302075 PMC3282876

[32] Codd V, Mangino M, van der Harst P, Braund PS, Kaiser M, Beveridge AJ, et al Common variants near TERC are associated with mean telomere length. Nat Genet. 2010;42(3):197–9. 10.1038/ng.532 20139977 PMC3773906

[33] Warnecke RB, Oh A, Breen N, Gehlert S, Paskett E, Tucker KL, et al Approaching Health Disparities From a Population Perspective: The National Institutes of Health Centers for Population Health and Health Disparities. American Journal of Public Health. 2008;98(9):1608–15. 10.2105/ajph.2006.102525 18633099 PMC2509592

[34] Paskett ED, McLaughlin JM, Reiter PL, Lehman AM, Rhoda DA, Katz ML, et al Psychosocial predictors of adherence to risk-appropriate cervical cancer screening guidelines: A cross sectional study of women in Ohio Appalachia participating in the Community Awareness Resources and Education (CARE) project. Preventive Medicine. 2010;50(1–2):74–80. 10.1016/j.ypmed.2009.09.001 19744509 PMC2813897

[35] Peek MK, Cutchin MP, Salinas JJ, Sheffield KM, Eschbach K, Stowe RP, et al Allostatic Load Among Non-Hispanic Whites, Non-Hispanic Blacks, and People of Mexican Origin: Effects of Ethnicity, Nativity, and Acculturation. American Journal of Public Health. 2010;100(5):940–6. 10.2105/ajph.2007.129312 19834005 PMC2853615

[36] Rebbeck TR, Rennert H, Walker AH, Panossian S, Tran T, Walker K, et al Joint effects of inflammation and androgen metabolism on prostate cancer severity. International Journal of Cancer. 2008;123(6):1385–9. 10.1002/ijc.23687 18566991 PMC2700293

[37] Krieger N, Smith K, Naishadham D, Harman C, Barbeau EM. Experiences of discrimination: validity and reliability of a self-report measure for population health research on racism and health. Social Science and Medicine. 2005;61:1576–96. 16005789 10.1016/j.socscimed.2005.03.006

[38] Cohen S, Kessler RC, Underwood L. Perceived stress scale Measuring stress: A guide for health and social scientists. Oxford University Press 1994;New York.

[39] Wang L, Liao W-C, Tsai C-J, Wang L-R, Mao IF, Chen C-C, et al The Effects of Perceived Stress and Life Style Leading to Breast Cancer. Women & Health. 2012;53(1):20–40. 10.1080/03630242.2012.732680 23421337

[40] Shell AM, Peek MK, Eschbach K. Neighborhood Hispanic composition and depressive symptoms among Mexican-descent residents of Texas City, Texas. Social Science & Medicine. 2013;99(0):56–63. 10.1016/j.socscimed.2013.10.006. 24355471 PMC3904495

[41] Radloff L. The CES-D scale: a self-report depression scale for research in the general population. Applied Psychological Measurement. 1977;1:385–401.

[42] Eaton WW, Smith C, Ybarra M, Muntaner C, Tien A. Center for Epidemiologic Studies Depression Scale: review and revision (CESD and CESD-R). In ME Maruish (Ed) The Use of Psychological Testing for Treatment Planning and Outcomes Assessment. 2004;3rd Edition(Volume 3: Instruments for Adults):363–77.

[43] Crockett LJ, Randall B, Shen YL, Russell ST, Driscoll AK. Measurement equivalence of the center for epidemiological studies depression scale for Latino and Anglo adolescents: a national study Journal of Consulting and Clinical Psychology. 2005;73(1):47–58. 15709831 10.1037/0022-006X.73.1.47

[44] Flores E, Tschann J, Dimas J, Bachen E, Pasch L, De Groat C. Perceived discrimination, perceived stress, and mental and physical health among Mexican-origin adults. Hispanic Journal of Behavioral Sciences. 2008;30(4):401–24.

[45] Kimura M, Stone RC, Hunt SC, Skurnick J, Lu X, Cao X, et al Measurement of telomere length by the Southern blot analysis of terminal restriction fragment lengths. Nat Protocols. 2010;5(9):1596–607. 10.1038/nprot.2010.124 21085125

[46] Weischer M, Nordestgaard BG, Cawthon RM, Freiberg JJ, Tybjærg-Hansen A, Bojesen SE. Short Telomere Length, Cancer Survival, and Cancer Risk in 47102 Individuals. Journal of the National Cancer Institute. 2013;105(7):459–68. 10.1093/jnci/djt016 23468462

[47] Codd V, Mangino M, van der Harst P, Braund PS, Kaiser M, Beveridge AJ, et al Common variants near TERC are associated with mean telomere length. Nat Genet. 2010;42(3):197–9. http://www.nature.com/ng/journal/v42/n3/suppinfo/ng.532_S1.html. 10.1038/ng.532 20139977 PMC3773906

[48] Codd V, Nelson CP, Albrecht E, Mangino M, Deelen J, Buxton JL, et al Identification of seven loci affecting mean telomere length and their association with disease. Nat Genet. 2013;45(4):422–7. http://www.nature.com/ng/journal/v45/n4/abs/ng.2528.html#supplementary-information. 10.1038/ng.2528 23535734 PMC4006270

[49] Maubaret CG, Salpea KD, Romanoski CE, Folkersen L, Cooper JA, Stephanou C, et al Association of TERC and OBFC1 Haplotypes with Mean Leukocyte Telomere Length and Risk for Coronary Heart Disease PLoS ONE. 2013;8(12):e83122 10.1371/journal.pone.0083122 24349443 PMC3861448

[50] Bojesen SE, Pooley KA, Johnatty SE, Beesley J, Michailidou K, Tyrer JP, et al Multiple independent variants at the TERT locus are associated with telomere length and risks of breast and ovarian cancer. Nat Genet. 2013;45(4):371–84. http://www.nature.com/ng/journal/v45/n4/abs/ng.2566.html#supplementary-information. 10.1038/ng.2566 23535731 PMC3670748

[51] Nordfjäll K, Eliasson M, Stegmayr B, Melander O, Nilsson P, Roos G. Telomere Length Is Associated With Obesity Parameters but With a Gender Difference. Obesity. 2008;16(12):2682–9. 10.1038/oby.2008.413 18820651

[52] Levy D, Neuhausen SL, Hunt SC, Kimura M, Hwang S-J, Chen W, et al Genome-wide association identifies OBFC1 as a locus involved in human leukocyte telomere biology. Proceedings of the National Academy of Sciences. 2010;107(20):9293–8. 10.1073/pnas.0911494107 PMC2889047 20421499

[53] Hunt SC, Chen W, Gardner JP, Kimura M, Srinivasan SR, Eckfeldt JH, et al Leukocyte telomeres are longer in African Americans than in whites: the National Heart, Lung, and Blood Institute Family Heart Study and the Bogalusa Heart Study. Aging Cell. 2008;7(4):451–8. 10.1111/j.1474-9726.2008.00397.x 18462274 PMC2810865

[54] Hubbard AE, Ahern J, Fleischer NL, Laan MVd, Lippman SA, Jewell N, et al To GEE or Not to GEE: Comparing Population Average and Mixed Models for Estimating the Associations Between Neighborhood Risk Factors and Health. Epidemiology. 2010;21(4):467–74 10.1097/EDE.0b013e3181caeb90 20220526

[55] Aubert G, Lansdorp PM. Telomeres and Aging. Physiol Rev. 2008;88(2):557–79. 10.1152/physrev.00026.2007 18391173

[56] Engelhardt M, Kumar R, Albanell J, Pettengell R, Han W, Moore MAS. Telomerase regulation, cell cycle, and telomere stability in primitive hematopoietic cells. Blood. 1997;90(1):182–93. 9207452

[57] Lynch SM, Major JM, Cawthon R, Weinstein SJ, Virtamo J, Lan Q, et al A prospective analysis of telomere length and pancreatic cancer in the alpha-tocopherol beta-carotene cancer (ATBC) prevention study. International Journal of Cancer. 2013;133(11):2672–80. 10.1002/ijc.28272 23674344 PMC5646275

[58] Rode L, Nordestgaard BG, Bojesen SE. Peripheral Blood Leukocyte Telomere Length and Mortality Among 64 637 Individuals From the General Population. Journal of the National Cancer Institute. 2015;107(6). 10.1093/jnci/djv074 25862531

[59] Ma H, Zhou Z, Wei S, Liu Z, Pooley KA, Dunning AM, et al Shortened Telomere Length Is Associated with Increased Risk of Cancer: A Meta-Analysis. PLoS ONE. 2011;6(6):e20466 10.1371/journal.pone.0020466 21695195 PMC3112149

[60] Hubbard AE, Ahern J, Fleischer NL, Van der Laan M, Lippman SA, Jewell N et al To GEE or not to GEE: comparing population average and mixed models for estimating the associations between neighborhood risk factors and health. Epidemiology 2010;21(4):467–74. 10.1097/EDE.0b013e3181caeb90 20220526

[61] National Research Council (US) Panel on Race E, and Health in Later Life. Critical Perspectives on Racial and Ethnic Differences in Health in Late Life. Washington (DC): National Academies Press (US); 2004 Available from: Available: http://www.ncbi.nlm.nih.gov/books/NBK25526/. 20669464

[62] Palloni A, Arias E. Paradox lost: explaining the Hispanic adult mortality advantage. Demography. 2004;41(385–418). 10.1353/dem.2004.0024 15461007

[63] Markides KS, Coreil J. The health of Hispanics in the Southwestern United States: an epidemiologic paradox. Public Health Reports 1986;101:253–65. 3086917 PMC1477704

[64] Markides KS, Eschbach K. Hispanic paradox in adult mortality in the United States In Rogers R & Crimmins E (Eds), International Handbook of Adult Mortality New York: Springer,. 2011:227–40.

[65] Bleil ME, Adler NE, Pasch LA, Sternfeld B, Gregorich SE, Rosen MP, et al Depressive symptomatology, psychological stress, and ovarian reserve: a role for psychological factors in ovarian aging? Menopause. 2012;19(11):1176–85 10.097/gme.0b013e31825540d8 22760086 PMC3465629

[66] Weng N-P. Interplay between telomere length and telomerase in human leukocyte differentiation and aging. Journal of Leukocyte Biology. 2001;70(6):861–7. 11739547

[67] Hunt SC, Chen W, Gardner JP, Kimura M, Srinivasan SR, Eckfeldt JH et al Leukocyte telomeres are longer in African Americans than in whites: the National Heart, Lung, and Blood Institute Family Heart Study and the Bogalusa Heart Study. Aging Cell. 2008;7(4):451–8. 10.1111/j.1474-9726.2008.00397.x 18462274 PMC2810865

