# Supplementary material for: Race, Ethnicity, Psychosocial Factors, and Telomere Length in a Multicenter Setting
Source: PLoS One. 2016 Jan 11;11(1):e0146723. doi: 10.1371/journal.pone.0146723 (PMC4709232; doi:10.1371/journal.pone.0146723)
Supplement: S1 Protocol — (DOCX) [file pone.0146723.s001.docx]

**Supporting Information**

**S1 Protocol.** **Review of Multilevel studies in Telomere Length.**

A manuscript review was conducted to identify epidemiologic, multicenter studies focused on factors affecting telomere length in adult populations. A multicenter study is defined here as a study comprised of individual research projects that collected data independently, then collaborated with other projects, and merged data to analyze telomere length as an overall, combined study outcome. Articles were excluded if only one university or academic center oversaw and was responsible for original data collection under a single study protocol, or if the main association analysis remained stratified by project or center. This is because the methodological concerns associated with varying population characteristics and laboratory methods are often minimized under these circumstances, and these studies have been reviewed previously [[4](#_ENREF_4), [5](#_ENREF_5), [10](#_ENREF_10), [11](#_ENREF_11)] .

A search of the electronic database, PubMed/Medline, was conducted in 2014. Studies from 2002 to the present were identified using the key words, “telomere length” paired with “multicenter(n=16)” or “consortium(n=12)” or “registry”(n=12). Citations in articles were cross-referenced to obtain additional sources. Study design, type of risk factor, or disease under study were not selection factors since the focus of this investigation is on methodological considerations in multicenter studies, not overall association findings. Eleven articles were retrieved and 9 met the criteria for inclusion listed below:

1. Research studies that involved adult, human participants and combined data from multiple centers with a primary aim of assessing the relationship between a risk factor and telomere length measured in blood.

2. The study reported clear methodologies for measuring telomere length.

3. It was written in English.

We summarized methodological approaches for each study based on laboratory, population factors, and statistical approaches known to affect telomere length measurements in literature[[4](#_ENREF_4), [5](#_ENREF_5), [10](#_ENREF_10), [11](#_ENREF_11), [20](#_ENREF_20)] (**S1 Table**).
