# Supplementary material for: Race, Ethnicity, Psychosocial Factors, and Telomere Length in a Multicenter Setting
Source: PLoS One. 2016 Jan 11;11(1):e0146723. doi: 10.1371/journal.pone.0146723 (PMC4709232; doi:10.1371/journal.pone.0146723)
Supplement: S2 Protocol — (DOCX) [file pone.0146723.s002.docx]

**S2 Protocol. Supplementary Laboratory Methods**

*Terminal Restriction Fragment (TRF) assay*: All assays were measured in a single laboratory at the Wistar Institute. Genomic DNA samples were digested with restriction enzymes Hinf I (10

U) and Rsa I (10

U; Roche), then the digested DNA samples (1-5

µg each) along with molecular weight DNA markers (1-kb DNA ladder plus λ DNA/Hind III fragments; Invitrogen, Carlsbad, CA) were resolved on 0.8% agarose gels and transferred to nitrocellulose membranes by southern blotting. Membranes were hybridized overnight using radioactively-labeled (TTAGGG) probes, and the radioactive signal was detected and digitized using a phosphorImaging system. The phosphorImager signals (adjusted for background) versus DNA migration distances were determined for each sample([39](#_ENREF_39)), and mean TRF in kb was determined using Telorun software([39](#_ENREF_39)). Each sample was run in duplicates on separate gels, and the average value in kb was used.

*Quantitative Telomere PCR (qPCR):* For a subset of Cross-Center samples (UPHS, n=101 and OSU, n=111), center samples were batch analyzed to minimize inter-assay variation. Each plate (384 wells on each plate) contained a set of standards spanning an 81-fold range prepared by serial dilution and analyzed in triplicate. Two master mixes of PCR reagents were prepared, one with the telomere primers (telc and telg) and the other with either the albumin (albd, and albu) or the beta-globin (hgbu, and hgbd) primers for single-copy reference genes. LTL did not vary by reference gene primer. The final primer concentrations in each 0.8X SYBR Green I Master Mix (Agilent Technologies) PCR reaction mix were: 900nM of the telomere pair, 900nM of the albumin pair, or 500nM of the beta-globin pair. The ABI software SDS version 2.0 was used to generate two standard curves from each plate, one for the telomere amplification, and the other for the single copy gene. The ratio (T/S) of the telomere copy number (T) to the single gene copy number (S) was generated for each experimental sample, and the value averaged across the triplicates.
