# Supplementary material for: Race, Ethnicity, Psychosocial Factors, and Telomere Length in a Multicenter Setting
Source: PLoS One. 2016 Jan 11;11(1):e0146723. doi: 10.1371/journal.pone.0146723 (PMC4709232; doi:10.1371/journal.pone.0146723)
Supplement: S1 Table — (DOCX) [file pone.0146723.s003.docx]

**S1 Table.** Evaluation and Summary of Methodologies employed in Multi-center ,Telomere Length(TL) Association Studies from 2002-Present

|  | **LABORATORY CONSIDERATIONS** | | | | | **POPULATION FACTORS** | | | | **STATISTICAL METHODS** | | | |  |
| --- | --- | --- | --- | --- | --- | --- | --- | --- | --- | --- | --- | --- | --- | --- |
|  | **# of Labs** | **DNA Source** | **DNA Extraction**  **Method(s)** | **Assay(s)^a^** | **Coefficient of (CV) Variation: reported range** | **Mean Age Range** | **Race** | **% men** | **Disease Status** | **Correlation w/ Age** | **Combined Telomere Outcome(s)** | **TL difference across centers/ labs** | **Statistical Approach** | **Findings** |
| **Studies by author** |  |  |  |  |  |  |  |  |  |  |  |  |  |  |
| Codd et al. March 2010  *(Combined 5 European cohorts)* | 5 | Blood Leukocyte | Puregene;  Phenol/ Chloroform; Qiagen^b^ | q-PCR (n=4)^c^ / Southern Blot(n=1) | Intra-assay: 1.5-2%  Inter-assay: 3.5-3.9% | 39.3-60.8 | White | 43 | Cardio-vascular/Renal Disease/ Healthy | Negative and Significant (assessed as linear regression) | Mean TL (T/S ratio or bp); Z-Score adjusted by age and sex | Yes (Mean TL range: 0.82-6.98) | 1. Linear Regression adjusted by age, center, sex; 2. Fisher Method; 3. Meta-analysis | Consistent across studies/various methods that 3q26 gene region (rs12696304) is associated w/ mean TL. |
| Codd et al. April 2013  *(Combined 21 European Cohorts)* | 5 | Blood Leukocyte | Puregene;  Phenol/ Chloroform; Qiagen^b^ | q-PCR ^c^ | Intra-assay:  2-5%  Inter-assay: 2.7-24.8% | 24-71.7 | White | 42 | Cardio-vascular Disease/ Smokers/ Healthy | Negative and Significant (assessed as T/S change per year) | Mean TL (T/S ratio or log-transformed T/S ratio); Z-Score adjusted by age and sex | Yes (Mean TL range: .004-3.71) | 1. Linear Regression adjusted by age, sex, family; 2. Meta-analysis | Study findings vary slightly by cohort, but loci associated with *TERC*, *TERT*, *NAF1*, *OBFC1* and *RTEL1 are* associated w/ mean TL. |
| Maubaret et al. 2013  *(Combined 5 European Case-control studies)* | 1 | Blood Leukocyte | Salting out | q-PCR (subset compared to Southern Blot for added quality control) | Intra-assay: 5% | 22.7-69.3 | White | 85 | Cardio-vascular disease or healthy | Not reported | Mean TL (bp calculated from T/S ratio vs Southern Blot plots) | Yes (Mean TL range: 6.80-9.82) | 1. Linear regression adjusted for age, center, gender,and physical activity; 2. Meta-analysis | Findings vary by study, but overall the previously reported association between *OBFC1* and TL confirmed |
| Bojesen et al. 2013  *(Combined 3 European cohorts)* | 2 | Blood Leukocyte | Phenol/ Chloroform; Qiagen^b^ | q-PCR | Not reported | 52-62 | White | 0 | Healthy controls | Not reported | Mean TL ( derived from Cycle threshold (CT) ) adjustments and base pair calculations from the T/S ratio) and relative change in mean TL per minor allele. | Yes. Lab difference assumed based on adjust-ment by plate and TL as a CT outcome. Center difference not reported | 1.Linear regression adjusted for age, plate, sex, principal component and study. 2. Meta-analysis | Findings vary by study in meta-analysis; TERT loci associated w/ longer TL (rs2736108 and rs7705526) |
| Cunningham et al., 2013  *(Combined international biobank, registry, and SPORE studies)* | 1 | Blood Leukocyte | Phenol-Chloroform, Puregene, Qiagen | q-PCR | Overall CV:  6% | 47-51 | Not re-ported | 52 | Colon cancer and healthy controls | Not reported | T/S ratio or log-transformed T/S ratio and differences in ratios between DNA extraction | Yes, based on DNA extraction | ANCOVA | RTL measured by Qiagen extracted DNA is smaller than other methods which could influence inconsistencies across studies |
| Weisher et al., 2012  *(Combined 2 European Cohorts)* | 1 | Blood Leukocyte | Phenol/ Chloroform; Qiagen^b^ | q-PCR | Overall CV: 9.3% | 56-68 | White | 46 | Healthy controls | Negative and Significant (assessed as linear regression) | Quartiles of Absolute TL base pairs (derived from Cycle threshold (CT) ) adjustments and base pair calculations from the T/S ratio). | Yes. Lab difference assumed based on adjust-ment by plate and TL as CT outcome. Center difference not reported. | Chi-square, Kruskal Wallis, p-trend treating TL as an ordinal variable. | TL is associated w/ age, bmi, male gender, smoking, alcohol intake. |
| Levy et al. 2010  *(Combined 4 US Cohorts and 1 European Twin Study)* | 1 | Blood Leukocyte | Qiagen, Phenol-chloroform, Puregene^b^ | Southern Blot | Not reported | 35-75 | White^e^  White/Black^f^ | 46 | Subjects from Heart Disease Studies (family/ popu-lation), but dis-ease rates not reported | Not reported | Mean TL (bp) | Not reported | Meta-analysis using Linear Regression and linear mixed effects regression (family studies) adjusted for age, age^2^,  sex, bmi, smoking. | TERC and OBFC1 associated with TL; Findings not reported by study. |
| Hunt et al. 2008  *(Combined 2 U.S. studies, one family and one population-based)* | 1 | Blood Leukocyte; T-cells and neutrophil | Qiagen, Phenol-chloroform, Puregene^b^ | Southern Blot w/ different restriction enzymes and using standard and overlay method(q-PCR on a subset) | Standard overall CV:  1.43%  Overlay overall CV:  2.40%  q-PCR: 6.40% | 19-93 | White and Black | 41 | Family and commun-ity based heart disease studies, but disease rates not reported | Significant and negative association with age in whites and blacks, but not by sex | Mean TL (calculated by standard and overlay method) adjusted for age and bmi; Difference in mean TL by race and gender | Not reported | Generalize estimating equations and ex-changeable correlation matrix comparing race-specific association of age with TL adjusted for sex/BMI | Sex- and BMI-adjusted TL became shorter with age at a steeper slope in blacks than in whites; Findings not reported by study. |
| Nordfjall et al. 2008  (Combined 2 European Cohorts) | 1 | Blood Leukocyte | Qiagen, phenol-chloroform, Puregene, Chemagen^b^ | q-PCR | Overall CV: 3.96% | 44.5-60.5 | White | 52 | Cancer and Cardio-vascular disease studies that include smokers, those w/ hyper-tension, etc. | Significant and Negative (assessed w/ correlation) | Log-transformed T/S ratio adjusted for age, sex, and center | No center differenceafter adjusting for age. | Analysis of covariance and correlation | TL is associated with an “obesity-phenotype” but only in women |

^a^ Quantitative Polymerase Chain Reaction(q-PCR) is often reported as a ratio of telomere length repeat length (T) to copy number of a single-copy gene or standard(S) DNA, called a T/S ratio; Southern blot assays report telomere length in terms of base pairs(bp).

^b^ Had to consult source manuscripts to identify potential sources of DNA extraction and did not find this information for each cohort in some instances. Also, for some cohorts, DNA extraction methods were different in two separate source manuscripts and it’s unclear which extraction method was used for the telomere length study.

^c^ Different DNA standards(S) were used to generate T/S ratios and were used to explain differences in mean T/S ratios across studies or cohorts.

^e^ In the genome-wide association study(GWAS), all were white participants(n=3417).

^f^ In the replication of the GWAS findings(n=1893), the study population included whites and blacks and findings differed slightly by race, which could have been due to the small sample size of blacks(n=574).
