# Supplementary material for: Race, Ethnicity, Psychosocial Factors, and Telomere Length in a Multicenter Setting
Source: PLoS One. 2016 Jan 11;11(1):e0146723. doi: 10.1371/journal.pone.0146723 (PMC4709232; doi:10.1371/journal.pone.0146723)
Supplement: S2 Table — (DOCX) [file pone.0146723.s004.docx]

**S2 Table.** Unadjusted Median and Mean LogTelomere Length(TL in kb) by Study Characteristics(No Cancer, n=1261)

|  | **Median TL (kb) (Interquartile Range)**^a^ | **p-value**^b^ | **Mean logTL(SD)** | **p-value**^c^ |
| --- | --- | --- | --- | --- |
| **Age** |  |  |  |  |
| Younger age(<=51) | 6.35(4.33-8.17) |  | 1.78(0.44) |  |
| Older age(>51) | 6.16(4.26-8.17) | 0.52 | 1.76(0.46) | 0.49 |
| **Gender** |  |  |  |  |
| Female | 6.33 (4.48-8.27) |  | 1.78(0.44) |  |
| Male | 6.01 (4.10-8.00) | 0.12 | 1.74(0.46) | 0.13 |
| **Race** |  |  |  |  |
| Non-Hispanic White | 5.96(4.24-7.92) |  | 1.73(0.44) |  |
| African American | 6.48(4.37-8.32) |  | 1.77(0.48) |  |
| Hispanic | 6.44 (4.42-8.32) | 0.03 | 1.79(0.45) | 0.08 |
| **Education** |  |  |  |  |
| > High School | 6.18(4.21-8.06) |  | 1.75(0.45) |  |
| High School/GED | 6.05(4.18-8.19) |  | 1.74(0.47) |  |
| <High School | 6.44(4.59-8.16) | 0.13 | 1.81(0.42) | 0.07 |
| **Perceived Stress** |  |  |  |  |
| High Stress | 6.16(4.23-8.17) |  | 1.76(0.45) |  |
| Low Stress | 6.38(4.37-8.15) | 0.29 | 1.78(0.45) | 0.72 |
| **Depression** |  |  |  |  |
| High Depression | 6.45(4.50-8.44) |  | 1.79(0.44) |  |
| Low Depression | 6.26(4.26-8.03) | 0.20 | 1.75(0.45) | 0.22 |

^a^ Medians(interquartile range for the median); ^b^ p-values comparing characteristics across 3 or more groups using Kruskal Wallis Test, otherwise used Wilcoxon Ranked Sum Test; ^c^ p-values comparing characteristics across 3 or more groups using ANOVA, otherwise used T-test.
